# International Overdose Awareness Day — August 31, 2017

**DOI:** 10.15585/mmwr.mm6634a1

**Published:** 2017-09-01

International Overdose Awareness Day is a global event held each year on August 31. This event aims to raise awareness that overdose deaths are preventable, reduce stigma about substance use disorders and drug-related deaths, highlight community drug-related services, and support evidence-based policies and programs to prevent or reduce drug-related harms. Additional information is available at https://www.overdoseday.com.

The opioid overdose epidemic resulted in the deaths of approximately 300,000 persons in the United States during 1999–2015, including 33,000 in 2015 (*1*). The first wave of deaths began in 1999 and included deaths involving prescription opioids (*1*). It was followed by a second wave, beginning in 2010, and characterized by deaths involving heroin (*2*). A third wave started in 2013, with deaths involving synthetic opioids, particularly illicitly manufactured fentanyl (IMF) (*3*). IMF is now being used in combination with heroin, counterfeit pills, and cocaine (*3*).

Reports in this issue of *MMWR* 1) highlight how increases in deaths and death rates related to heroin and synthetic opioids mirror data tracking illicit drugs and 2) describe the role of IMF and fentanyl analogs in 281 overdose deaths in 2 months in Ohio. Additional data and information on CDC’s state-level efforts to address drug-related deaths are available at https://www.cdc.gov/drugoverdose/index.html.
